# Different walls for rods and balls: the diversity of peptidoglycan

**DOI:** 10.1111/mmi.12513

**Published:** 2014-01-27

**Authors:** Robert D Turner, Waldemar Vollmer, Simon J Foster

**Affiliations:** 1 The Krebs Institute, Department of Molecular Biology and Biotechnology, Firth Court, Western Bank, The University of SheffieldSheffield, S10 2TN, UK; 2 The Centre for Bacterial Cell Biology, Baddiley Clark Building, Medical School, Newcastle UniversityRichardson Road, Newcastle upon Tyne, NE2 4AX, UK

## Abstract

Peptidoglycan performs the essential role of resisting turgor in the cell walls of most bacteria. It determines cell shape, and its biosynthesis is the target for many important antibiotics. The fundamental chemical building blocks of peptidoglycan are conserved: repeating disaccharides cross-linked by peptides. However, these blocks come in many varieties and can be assembled in different ways. So beyond the fundamental similarity, prodigious chemical, organizational and architectural diversity is revealed. Here, we track the evolution of our current understanding of peptidoglycan and underpinning technical and methodological developments. The origin and function of chemical diversity is discussed with respect to some well-studied example species. We then explore how this chemistry is manifested in elegant and complex peptidoglycan organization and how this is interpreted in different and sometimes controversial architectural models. We contend that emerging technology brings about the possibility of achieving a complete understanding of peptidoglycan chemistry, through architecture, to the way in which diverse species and populations of cells meet the challenges of maintaining viability and growth within their environmental niches, by exploiting the bioengineering versatility of peptidoglycan.

## Introduction

**Figure 1:**
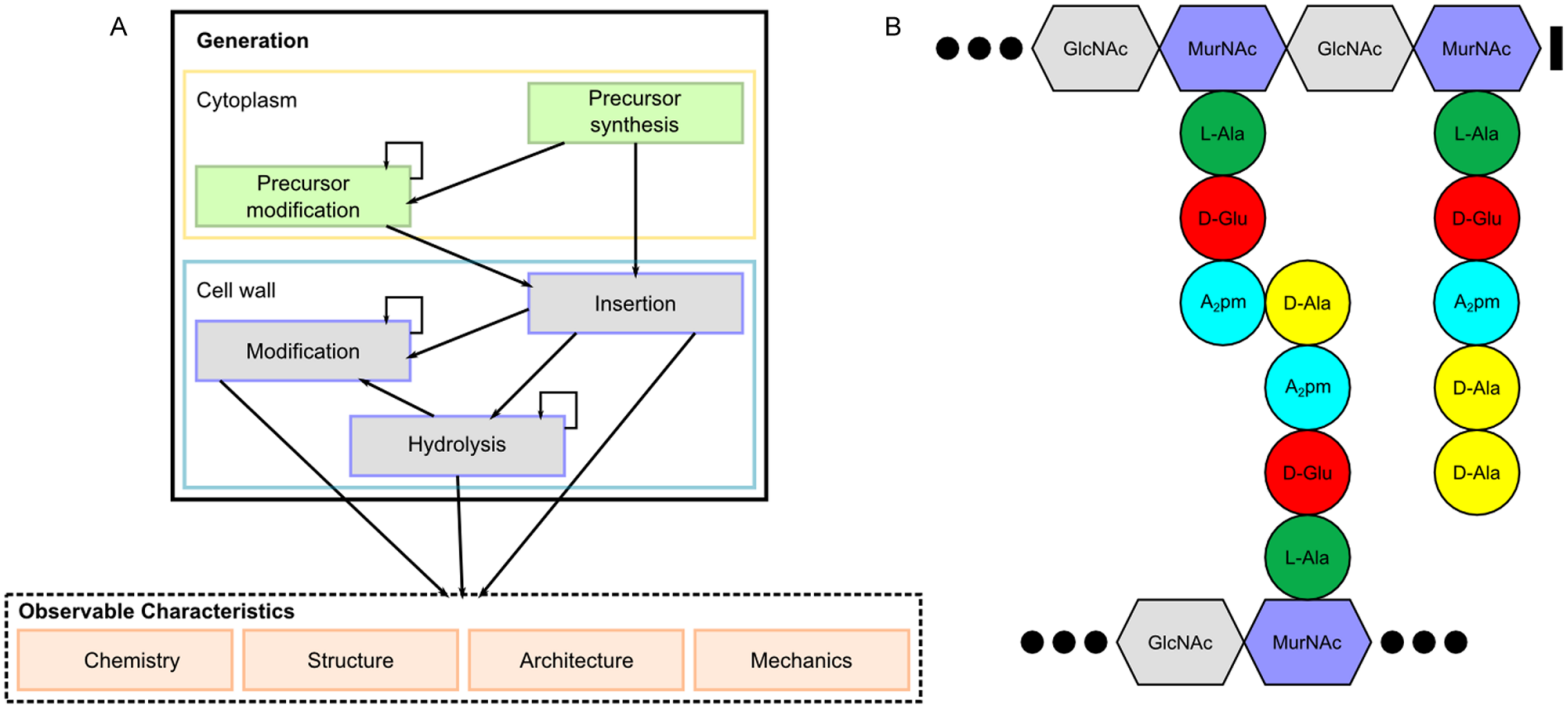
A. The general process by which peptidoglycan is generated. Broadly conserved precursors may be modified and are then inserted into the sacculus, which can be modified and/or hydrolysed resulting in a diversity of measurable characteristics. B. Simplified peptidoglycan chemistry pertaining to many Gram-negative and some Gram-positive species. GlcNAc, *N*-acetylglucosamine; MurNAc, *N*-acetylmuramic acid; L-Ala, L-Alanine; D-Glu, D-Glutamic acid; A_2_pm, meso-Diaminopimelic acid; D-Ala, D-Alanine.

Peptidoglycan is a component of nearly all bacterial cell walls and has a variety of functions. It is a huge single molecule (sacculus) surrounding the cytoplasmic membrane, and as such it enables the cell envelope to resist the turgor resulting from the difference in composition between the cytoplasm and the external environment. Furthermore, peptidoglycan is the anchoring point for proteins and other polymers. Another common function is interaction with a host: fragments of peptidoglycan shed by commensal or pathogenic bacteria being recognized by the immune system. The observed characteristics of peptidoglycan (chemistry, structure, architecture and mechanical properties) are the result of a series of possible reactions which can occur both in the cytoplasm and in the cell wall itself (Fig. 1A).

The basic peptidoglycan precursor is broadly conserved across species, consisting of disaccharides of *N*-acetylglucosamine and *N*-acetylmuramic acid carrying short peptides (Fig. 1B), but the chemistry and architecture of the final peptidoglycan cell wall are diverse. High performance liquid chromatography (HPLC) analysis has revealed a great chemical repertoire of peptidoglycan subunits even within an isogenic population (Vollmer *et al*., 2008a; Desmarais *et al*., 2013). Numerous studies have shown large scale ‘architectural’ features comprised of peptidoglycan on the surface of living cells, or as constituents of sacculi, and these differ between species and stages in the cell cycle (Amako *et al*., 1982; Touhami *et al*., 2004; Plomp *et al*., 2007; Hayhurst *et al*., 2008; Andre *et al*., 2010; Turner *et al*., 2010; 2013,; Wheeler *et al*., 2011). These features range in size from tens of nanometers to micrometers. Peptidoglycan polymers are arranged to fulfil their functions on a length scale between that of these features and the subnanometer scale of muropeptide chemistry. Recently, application of techniques such as Electron Cryo Tomography (ECT) and Atomic Force Microscopy (AFM) have made molecular organization on these length scales easier to address directly (Li and Jensen, 2009; Dupres *et al*., 2010).

Others have reviewed peptidoglycan chemistry and structural models (Vollmer *et al*., 2008a; Vollmer and Seligman, 2010; Desmarais *et al*., 2013), chemical modification of peptidoglycan (Vollmer, 2008) and the role of regulatory proteins and mechanical forces in peptidoglycan synthesis and hydrolysis (Typas *et al*., 2012). Here, we do not re-iterate this, neither do we discuss models of cell growth, except where particularly relevant. Instead, we illustrate the diversity of peptidoglycan structure, chemistry and architecture. We discuss the genesis of current models of peptidoglycan arrangement and how they have evolved with new data. Rather than try to cover every species, we will discuss the cases of *Staphylococcus aureus*, *Bacillus subtilis* and *Escherichia coli* in detail as these have been most heavily studied.

## A time traveller's guide to peptidoglycan structure

**Figure 2:**
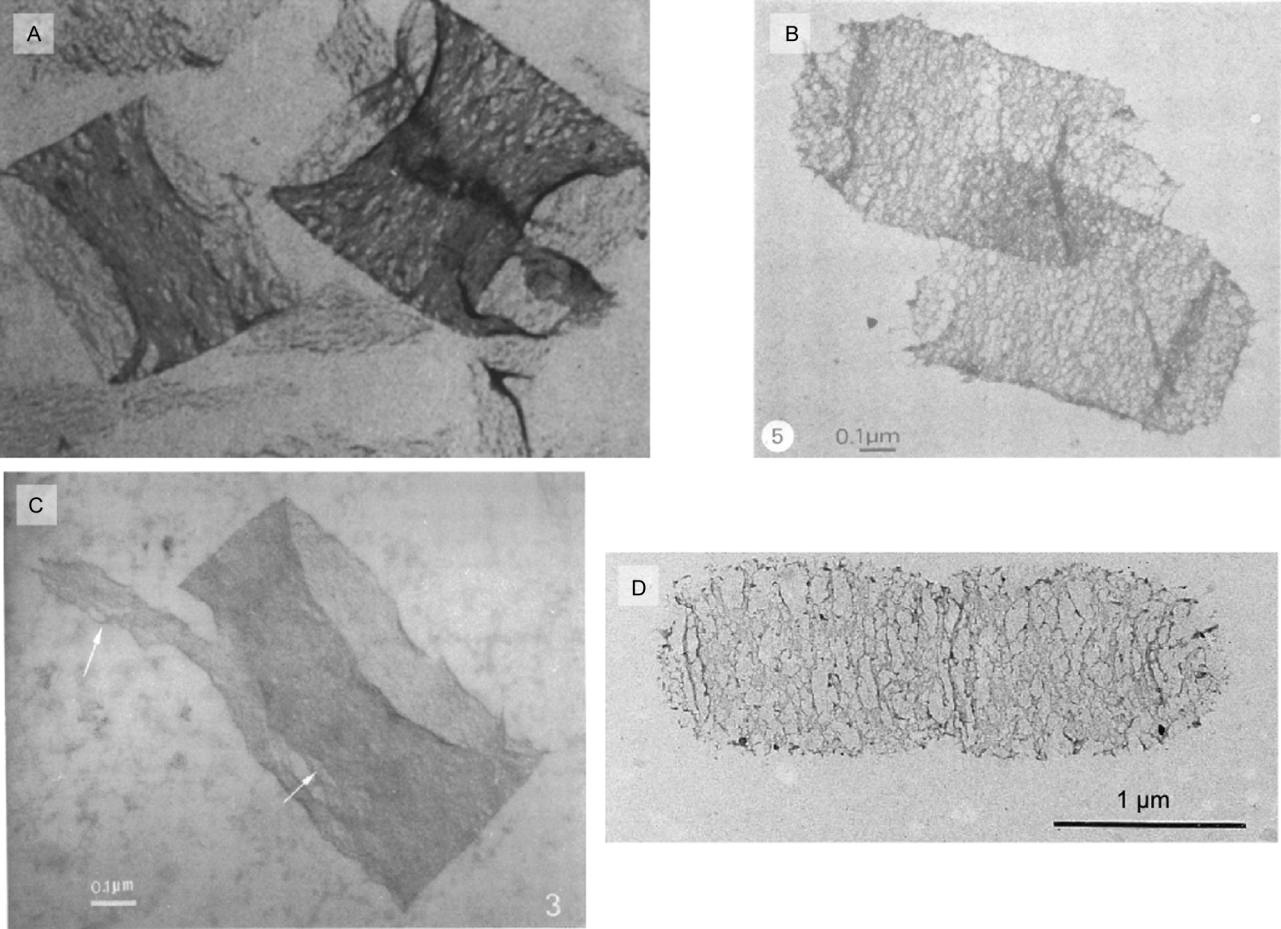
TEM images of sacculi.
*B. subtilis*, sacculi broken by sonication, teichoic acids extracted, 37 000× magnification (Verwer and Nanninga, 1976).*E. coli* partially digested with an endopeptidase breaking inter-peptide bonds (Verwer *et al*., 1978).*E. coli* broken by sonication (Verwer *et al*., 1980).*E. coli* partially digested with Cellosyl breaking intra-glycan bonds (de Pedro *et al*., 1997). A partial digestion with lysozyme (also breaks intra-glycan bonds) had previously been reported to leave no oriented features.

Most of the structural work on peptidoglycan pertains to *E. coli*. Planar (glycan strands running parallel to the membrane) and perpendicular (much like the later scaffold model) models were first articulated in the early 1970s (Braun *et al*., 1973). Three assumptions were presented that should hold unless contradicted by experimental evidence. Briefly, these were (i) peptidoglycan has a regular structure, (ii) polysaccharide chains run in parallel, and (iii) the spatial relationship between peptide side-chains is constant so that biosynthetic enzymes are presented with a consistent stereochemistry. On the basis of these assumptions and the measured thickness of the peptidoglycan layer in *E. coli*, a planar crystalline model was adopted with the caveat that there would also be less dense arrangements present in the sacculus between the crystalline areas. The model accounted for an experimentally determined 15–30% cross-linking of peptides, all of which were placed in-plane, but at the time no information on glycan strand length was available. It was proposed that this model could be extended to all Gram-negatives and many layers of this material could stack up to form Gram-positive peptidoglycan. Subsequent electron microscopy of *B. subtilis* sacculi showed circumferential features, too large to be glycans, but which were explained in terms of circumferential orientation of glycans (Fig. 2A, Verwer and Nanninga, 1976). This model is still widely accepted and can be found in textbooks, despite many subsequent discoveries.

Shortly after the planar crystalline model was proposed, a pair of thorough X-ray scattering studies of many organisms including *E. coli*, *B. subtilis* and *S. aureus* gave a strong indication that peptidoglycan is not a crystalline material (Burge *et al*., 1977a,b,). The main periodicity identified was associated with the helical periodicity of the glycans such that disaccharide motifs have a spacing of 0.98 nm. A helical arrangement with fourfold symmetry was proposed for the glycans such that alternate peptides would be parallel, then perpendicular to a plane tangential to the cell surface. As a result of this, only half of the peptides might be in-plane and available to form cross-links in a single layered sacculus.

The X-ray scattering evidence sparked debate about the level of order present in peptidoglycan and one might have thought that direct measurement of strand organization through imaging would resolve this. However, Transmission Electron Microscopy (TEM) of negatively stained *E. coli* sacculi did not reveal direct evidence of glycan strand arrangement. Early experiments in which sacculi were partially digested with enzymes targeting specific bonds (Fig. 2B), or broken by sonication prior to imaging (Fig. 2C), led to the conclusion that glycan strands were circumferentially oriented (Verwer *et al*., 1978; 1980). However, conflicting evidence was obtained in later studies (de Pedro *et al*., 1997; Koch, 1998 – compared Fig. 2B and Fig. 2D), which show similar effects from enzymes targeting different bonds. Nonetheless, when AFM measurements on sacculi showed a higher elastic modulus (i.e. lower elasticity) in the circumferential direction than the longitudinal axis, this was taken to be evidence for circumferential orientation of glycans (Yao *et al*., 1999).

**Figure 3:**
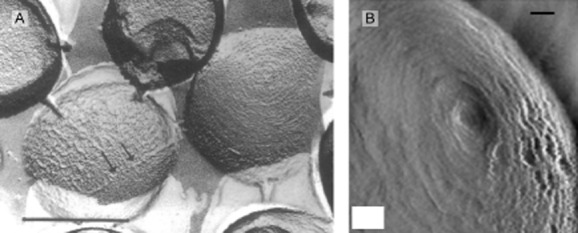
Freeze etch SEM (A) and AFM (B) images of the surface of intact *S. aureus*. Scale bars: (A) 500 nm; arrows indicate thick boundary line, (B) 50 nm (Amako *et al*., 1982; Touhami *et al*., 2004).

Concurrently with some of this work, freeze etch electron microscopy revealed surprising surface features associated with the peptidoglycan of *S. aureus*, suggesting a far more complex and heterogeneous arrangement of peptidoglycan than had been proposed previously (Fig. 3A, Amako *et al*., 1982); work later validated by AFM imaging of the surface of living and dividing cells (Fig. 3B, Touhami *et al*., 2004).

## The dawn of the muropeptide

Although pioneering work had revealed the peptidoglycan precursor (Park and Johnson, 1949) and established the biochemical diversity of peptidoglycan (Schleifer and Kandler, 1972), prior to 1988 our knowledge of peptidoglycan chemistry in the sacculus was largely derived from biochemical techniques that addressed the entire population of muropeptides at once. For example, one could analyse the amino acids/sugars and small fragments released by partial or complete hydrolysis of sacculi. HPLC muropeptide analysis revolutionized the study of peptidoglycan by enabling the muropeptide population to be separated based on chemical properties (Glauner *et al*., 1988). This meant that composition could be addressed in terms of larger building blocks i.e. those manipulated by penicillin-binding proteins (PBPs), hydrolases and other enzymes that act within the cell wall, rather than the cytoplasmic raw materials. By combining HPLC with genetic knockouts or digestion of sacculi with recombinant enzymes, it has been possible to assign roles to many of the genes involved in peptidoglycan biochemistry. On the basis of this knowledge, it is generally possible to look at a muropeptide profile and postulate how a particular muropeptide came to be. For example, a disaccharide–tetrapeptide monomer in *B. subtilis* is presumably the work of DacA, which removes the terminal D-alanine (Atrih *et al*., 1999), or of an endopeptidase cleaving in DD-cross-links.

Reverse phase HPLC requires that peptidoglycan is extracted from a (rather large) population of bacteria, digested with an enzyme, reduced and pumped through a column which separates the resulting fragments based on their chemical properties with a strong emphasis on hydrophobicity (Desmarais *et al*., 2013). The result is a population average, and there is always some material in the sample that cannot be resolved by the column. However, on the basis of this kind of data it is clear that while the muropeptides have a common precursor they are wildly diverse between species, within a sacculus and across a population. This is true of *S. aureus* (de Jonge *et al*., 1992), *B. subtilis* (Atrih *et al*., 1999) and *E. coli* (Glauner *et al*., 1988).

Peptidoglycan is often compared using the metrics of degree of cross-linking [i.e. (1/2 × Σdimers + 2/3 × Σtrimers + 3/4 × Σtetramers)/all muropeptides)] and glycan strand length. A range of cross-linking degrees have been measured for a variety of *E. coli* strains from 31–61% (Vollmer and Seligman, 2010). This is consistent with the degree of cross-linkage proposed in earlier structural models (Burge *et al*., 1977a, 1977b,). For exponentially growing *B. subtilis* it is 56%, in stationary phase 64% and in spores a remarkably low 3% (Atrih *et al*., 1996; 1999,). In *S. aureus* strains it varies between the high values of 74–92% (Snowden and Perkins, 1990; de Jonge *et al*., 1992; Boneca *et al*., 2000).

Glycan strand length can be analysed by determining the proportion of anhydro-disaccharide-containing muropeptides within a sample of muramidase digested material (Glauner *et al*., 1988). In several species, mostly Gram-negatives, these muropeptides indicate strand ends, so a higher proportion means shorter strands. Alternatively, glycan chains can be released from the peptides by an amidase and separated by reverse phase HPLC (Harz *et al*., 1990; Boneca *et al*., 2000) or size exclusion HPLC (Hayhurst *et al*., 2008). As with muropeptide profiles, strand length differs between species. In *E. coli*, average strand lengths between 18 and 60 disaccharides have been reported (Vollmer *et al*., 2008a). In *S. aureus*, strands have on average 6 disaccharides (Boneca *et al*., 2000). In *B. subtilis* some glycan strands are too long to be resolved by HPLC, in this case Atomic Force Microscopy (AFM) was applied to measure strands (Hayhurst *et al*., 2008). In this species they are up to 5 μm (corresponding to ∼5000 disaccharide units), and in some species of ovococci (Wheeler *et al*., 2011; Bui *et al*., 2012) there are strands in excess of 100 disaccharides in length (exact lengths not determined). Notably in *Helicobacter pylori* glycan strands are short: many strands have 6 disaccharides or less (Chaput *et al*., 2007).

## Vive la différence: explaining the diversity of peptidoglycan chemistry

Peptidoglycan monomers are synthesized as lipid-linked precursors (lipid II) which are flipped from the inner to the outer leaflet of the cytoplasmic membrane (Mohammadi *et al*., 2011) before being inserted into the pre-existing sacculus by PBPs. These monomers are disaccharide–pentapeptides, with interspecies differences in peptide composition. Chemical modification of peptidoglycan has been covered thoroughly in the literature and illustrative details can be found in recent reviews (Vollmer, 2008; Vollmer *et al*., 2008a,b,). In general, many Gram-positive Firmicutes have an L-lysine in position 3, whereas most Gram-negative proteobacteria have a *meso*-diaminopimelic acid. *Bacillus* and *Clostridia* species are notable exceptions to this, having Gram-negative style *meso*-diaminopimelic acid which, unlike in Gram-negatives, becomes amidated in most of the subunits (Vollmer *et al*., 2008a). The second amino group on the L-lysine or *meso*-diaminopimelic acid residue enables cross-linking via an amide bond to another peptide. This is not a universal mechanism and there are other forms of cross-linking. For example in some species, notably among the staphylococci and ovococci, cross-links are via an additional peptide bridge (Vollmer *et al*., 2008a). Even before lipid II is flipped, the precursor can be subject to modification. Important modifications to *S. aureus* lipid II are (i) the addition of the pentaglycine ‘branch’ by three Fem-type amino acid transferases that are critical, as *S. aureus* is unable to form direct cross-links between the peptides (Rohrer *et al*., 1999; Hübscher *et al*., 2007) and (ii) the amidation of glutamic acid by the amidotransferase MurT/GatD to form glutamine, that is essential in *S. aureus* (Figueiredo *et al*., 2012; Münch *et al*., 2012) and *S. pneumoniae*; in the latter the PBPs require glutamine residues to perform cross-linking reactions (Zapun *et al*., 2013).

Typically the initial cross-linking reaction liberates the terminal D-alanine of the donor molecule creating a penta-tetrapeptide containing dimer, a penta-tetra-tetrapeptide containing trimer or related higher cross-linked multimers. So in general at this stage there are few possible muropeptide products: an un-cross-linked pentapeptide monomer and cross-linked species with penta and tetrapeptides, and such peptides are seen in HPLC muropeptide analyses. However we have discussed that, for example in *S. aureus*, incomplete glutamic acid amidation and addition of glycines means that there are already many more possible muropeptides. This small set of muropeptides are the substrates for a battery of enzymes which can chemically modify peptidoglycan or break internal bonds (peptidoglycan hydrolases). The action of these enzymes results in further muropeptide diversity.

In *S. aureus*, a major chemical modification that occurs in the cell wall is *O*-acetylation of *N*-acetylmuramic acid (Bera *et al*., 2005), resulting in muramic acid which is both *N*-and *O*-acetylated. Catalysed by the integral membrane protein OatA, this modification contributes to lysozyme resistance.

In *B. subtilis*, major modifications are de-*N*-acetylation of *N*-acetylglucosamine and *N*-acetylmuramic acid (Zipperle *et al*., 1984; Atrih *et al*., 1999), and alteration of *N*-acetylmuramic acid to contain a δ-lactam ring in spore cortex peptidoglycan (Atrih *et al*., 1996; Popham *et al*., 1996). De-*N*-acetylation leads to increased resistance to lysozyme. One enzyme that executes de-*N*-acetylation is PdaA (Blair and van Aalten, 2004; Fukushima *et al*., 2005), which presumably acts in the cell wall rather than the cytoplasm (Vollmer and Tomasz, 2000). δ-lactam formation requires prior de-*N*-acetylation by PdaA and is executed by CwlD, presumably in the spore cortex (Sekiguchi *et al*., 1995; Gilmore *et al*., 2004).

In *E. coli*, as with many Gram-negative bacteria, glycan strands are terminated with 1,6-anhydromuramic acid (Höltje, 1998), likely to be largely a result of lytic transglycosylase activity (Höltje *et al*., 1975).

Peptidoglycan hydrolases are the remaining factors in generating peptidoglycan diversity. This area has been reviewed thoroughly (Vollmer *et al*., 2008b; van Heijenoort, 2011) and while *B. subtilis*, *S. aureus* and *E. coli* have different palettes of enzymes, they are able to act on a similarly broad range of cleavage sites. Hydrolases have diverse functions and affect muropeptides primarily by splitting and truncating peptide side-chains; they affect glycan strand length by creating shorter strands.

In general, it is possible to interpret all of the peaks in a HPLC muropeptide profile in terms of a history of sequential activities of the types of enzyme discussed here. However, despite this knowledge we are generally unable to assign muropeptides to particular subcellular locations, stages in the cell cycle or members of a population, due to the sample-averaging nature of HPLC.

## Scaffold model controversy

By the mid-1990s, much was known about muropeptide chemistry and glycan strand length had been determined in some organisms, but orthodox models of peptidoglycan structure were open to challenge. A neutron scattering study provided evidence that *E. coli* peptidoglycan has more than one layer (Labischinski *et al*., 1991), although this is disputed in light of ECT images (Gan *et al*., 2008). In a testament to the scarcity and conflicting nature of structural data, there was sufficient ambiguity over peptidoglycan structure that a scaffold model in which glycan strands are oriented perpendicular to the membrane (Dmitriev *et al*., 2003; 2004,) could be proposed. This could be rapidly discounted for *E. coli* (Vollmer and Höltje, 2004) on the basis that the average glycan strand is too long to fit within the periplasm if oriented perpendicular to the membranes (Harz *et al*., 1990). However, in some Gram-positive bacteria (including *S. aureus*) the cell wall is thick enough to accommodate the known length of the glycan strands in this orientation, leading to a scaffold interpretation of NMR data derived from small, soluble synthetic peptidoglycan fragments (Meroueh *et al*., 2006).

## Imaging architecture: the direct approach

The peptidoglycan layer is the main determinant of bacterial shape. When sacculi are purified, they retain morphological features such that it is easy to deduce the approximate overall shape of the living cell. In addition to this gross morphology, it is well established by decades of electron microscopy that subcellular ‘architectural’ features can be observed in some species. It is recent direct evidence, largely from ECT and AFM that currently shapes our understanding of peptidoglycan structure, either by revealing strand orientation directly or by revealing architectural features from which structure may be inferred. A major current challenge is visualization of both glycan strands and cross-links within sacculi – this has never been achieved.

## B*. subtilis*

Rod-shaped cells of *B. subtilis* grow by elongation of the cylindrical part of the cell followed by septation. Peptidoglycan is inserted in an apparent helical pattern during elongation (Daniel and Errington, 2003; Tiyanont *et al*., 2006). The insertion pattern during septation has not been determined. *B. subtilis* is also capable of sporulation, which follows asymmetric septation.

**Figure 4:**
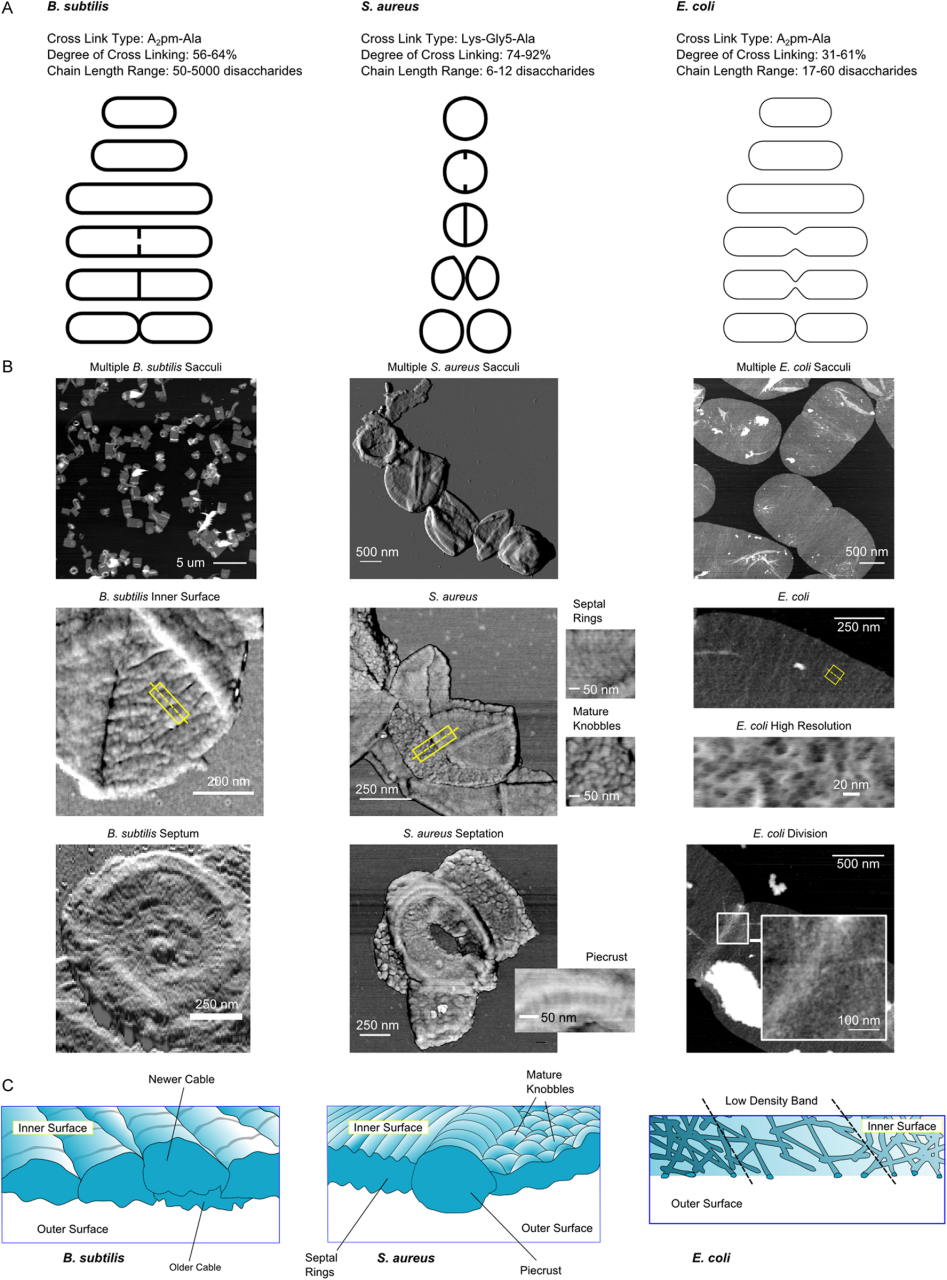
Peptidoglycan architecture in *B. subtilis*, *S. aureus* and *E. coli*.
Metrics of peptidoglycan for comparison. Ranges are lowest and highest values identified in the literature (Vollmer and Seligman, 2010; Wheeler *et al*., 2011). In *S. aureus* and *E. coli* these are average values, in *B. subtilis* they are a representative of the overall range.AFM gallery of sacculi comprising images comprising multiple sacculi per field, and key architectural details specific to each species (Hayhurst *et al*., 2008; Turner *et al*., 2010; 2013).Interpretive diagrams drawn from yellow rectangles marked in ‘B’.

Peptidoglycan organization in *B. subtilis* had long been assumed to take the form of layers of material where glycan strands run parallel to the membrane and circumferentially relative to the cell (Koch, 1995). AFM observations (Fig. 4B, Hayhurst *et al*., 2008) of the inner and outer surfaces of sacculi showed that in the cylindrical body of the cell fat, discontinuous bands of material much wider than glycans run circumferentially (Fig. 4B). In some cases of broken sacculi these bands remained intact and formed links between sections of sacculi. In the septum, a different architecture was observed: a thick outer band surrounded a spiral or centripetal rings on the septal disc itself (Fig. 4B). Higher resolution imaging of the bands seen in the cylindrical body revealed cross striations with an approximately 25 nm periodicity. This led to the interpretation that each band was a flattened cable of peptidoglycan (Fig. 4C). Partial hydrolysis of outer surface material was put forward for an explanation of why the cables appeared more distinct on the inside of the sacculus (more hydrolysis occurs at the outer surface (Smith *et al*., 2000). The cable concept indicates that the very long (>5 μm) glycan strands present in this species are packaged in such a way that they could be inserted in short helical segments with a length less than that of the circumference of the cell (Fig. 4B). A proposal was made that ropes consisting of a few glycans are coiled into the cables in order to package the long strands, however definitive determination of the internal structure is not possible without a means of direct visualization.

The features clearly visible in AFM images of the cylindrical part of *B. subtilis* sacculi and interpreted as cables are not apparent in electron cryo-microscopy (ECM) or ECT images of the cell wall or of sacculi (Matias and Beveridge, 2005; Beeby *et al*., 2013). Contrast (signal-to-noise ratio) limitations may make it difficult to visualize any internal organization of peptidoglycan in *B. subtilis* using these techniques. Nonetheless, peptidoglycan thickness was observed not to be uniform and rough textured (perhaps circumferential) outer surface features were visible in ECT images. Some of these appear superficially similar to those seen by AFM and may be related to those observed previously by TEM (Fig. 2A, Verwer and Nanninga, 1976).

The lack of direct visualization of the internal structure of the proposed cables and the absence of distinct cables in ECM and ECT images has led to an argument that they do not exist (Beeby *et al*., 2013). Molecular dynamics simulations comparing planar and scaffold models show that the planar model agrees best with the way in which peptidoglycan fragments fold after breaking of sacculi, as visualized by ECT. However, it is not clear how such a model would accommodate available AFM data, particularly the evidence for very long glycan strands.

## S*. aureus*

The presence of heterogeneity in the surface structures of *S. aureus* is very well established. In some strains the peptidoglycan is relatively well exposed and unobscured by capsular material or appendages (although still containing teichoic acids). Images of freeze etched *S. aureus* revealed two distinct surface textures demarked by thick lines (Amako *et al*., 1982; Giesbrecht *et al*., 1998). The surface textures were recapitulated in AFM images of living cells (Touhami *et al*., 2004) and ultimately determined to be comprised of peptidoglycan (Turner *et al*., 2010). One of these textures is described as either concentric rings or spirals, the other as rough or knobbly.

*S. aureus* is a nominally spherical organism that lays down a division septum to create two hemispherical daughter cells. These then grow into spheres and the process is repeated on orthogonal planes. Peptidoglycan insertion apparently occurs only during septation (Pinho and Errington, 2005) and is therefore associated with the ring or spiral surface architecture (Fig. 4B). The volume of the septal disc of peptidoglycan is lower than that of the hemispherical shell which it becomes, so the material must rearrange and reduce in density to take up this new shape as the cell expands. Mature peptidoglycan has a knobbly surface, so the peptidoglycan in the rings becomes rearranged into knobbles as expansion takes place (Fig. 4B). We hypothesize that this growth is facilitated by the action of peptidoglycan hydrolases, some of which are known to be septally located (Yamada *et al*., 1996), and by turgor pressure induced strain. Separating the septal and mature regions of peptidoglycan is a thick line of material, which when visualized by AFM appeared to have a ‘piecrust’ surface texture (Fig. 4B). These piecrust structures are bisected during consecutive rounds of division and are posited to have a role in establishing cell polarity (Turner *et al*., 2010).

While there is a wealth of direct evidence from microscopy illustrating the surface of *S. aureus* peptidoglycan, no direct visualization of the interior has been possible. Evidence from solid state NMR indicates that despite the quite disorganized surface, regions exhibiting a high level of local order are nonetheless present within the material (Kim *et al*., 2013). Perhaps hydrolases act between these highly ordered regions and the regions themselves are associated with the observed surface knobbles. Alternatively, the presence of ordered regions might be explicable in terms of nascent peptidoglycan having a different level of order compared with mature material.

There is no evidence from the study of sacculi to suggest any overall glycan strand orientation which might validate the broadly applied planar or scaffold models of peptidoglycan structure (Dmitriev *et al*., 2004) for this species of bacteria.

## E*. coli*

As a Gram-negative bacterium, *E. coli* has a much thinner layer of peptidoglycan than *S. aureus* or *B. subtilis* (Vollmer and Seligman, 2010). This has made it more tractable to high resolution imaging techniques in terms of determination of internal structure. ECT of *E. coli* sacculi revealed ‘tubes of density mostly in the plane of the sacculus and roughly perpendicular to the long axis of the cell’ (Gan *et al*., 2008). These were interpreted to be glycan strands as they were, in general, longer than would be expected for peptide cross bridges which are the other structures one might expect to see. This demonstrated that the scaffold model is absolutely not applicable to Gram-negatives (Dmitriev *et al*., 1999), which backs up the argument that glycan strands are too long to fit within the Gram-negative cell wall if arranged perpendicular to the membranes (Vollmer and Höltje, 2004). ECT data indicates that peptidoglycan organization is quite uniform across the sacculus.

Recent AFM of *E. coli* sacculi (Turner *et al*., 2013) supports many of the conclusions drawn from ECT. However, high resolution AFM images and *in situ* imaging of digestion of sacculi revealed features running parallel to the plane of the sacculus, but in many directions relative to its long axis. Also observed were bands of porosity running roughly circumferentially around the sacculi (Fig. 4B). This resulted in three key differences in conclusions: (i) glycans have no overall orientation relative to the cell, (ii) peptidoglycan is organized into bands of greater or lesser porosity which have an overall circumferential orientation, and (iii) thickness varies across the sacculus. These bands were proposed to define regions more or less available for insertion of new peptidoglycan, which is dependent on interactions between Lpo proteins in the outer membrane and PBPs in the inner membrane (Paradis-Bleau *et al*., 2010; Typas *et al*., 2010). It has been hypothesized that the activation of PBPs by outer-membrane proteins is a means to make peptidoglycan synthesis responsive to the properties of the pores (Turner *et al*., 2013) in the peptidoglycan, thereby coupling peptidoglycan growth with cell growth (Typas *et al*., 2012).

## Septation

Septation is a different process in each of our three examples and this has strong implications for peptidoglycan architecture. In *S. aureus* and vegetative *B. subtilis* it follows a pattern common to Gram-positives in that a septal disc is produced and then severed to liberate daughter cells (Fig. 4A). However, in *S. aureus* septal disc formation is preceded by formation of thick band of peptidoglycan dubbed the ‘piecrust’ for its often crimped surface texture. This is absent in *B. subtilis* septa (compare panels in Fig. 4B). In *S. aureus* expansion of the severed septal disc is thought to be the sole mechanism of cell growth (Pinho and Errington, 2005; Turner *et al*., 2010). In vegetative *B. subtilis* the septal disc matures to form a cell pole.

In *E. coli* septation is a very different process. The cell constricts due to the co-ordinated synthesis and cleavage of septal peptidoglycan before forming two fully mature opposing poles (Fig. 4A; de Pedro *et al*., 1997; Varma *et al*., 2007; Uehara *et al*., 2010). *E. coli* septal and polar peptidoglycan have not yet been studied in detail by AFM or ECT. However, AFM does reveal a thickening of the peptidoglycan at the leading edge of septum formation (Fig. 4B).

## Why and how is peptidoglycan so chemically diverse?

Our three case studies illustrate how diverse peptidoglycan architecture is across different species, and how much is still unknown about the way the sacculus is structured. However, there are even more unknowns when it comes to the way in which the sacculus is constructed and how the diversity of chemical motifs within a population of a single species maps onto architecture. As we have seen, we can explain how muropeptides have come about in terms of enzymatic pathways. We can also explain why some of these are present, e.g. *O*-acetylation in *S. aureus* imbues lysozyme resistance. However, many of the motifs revealed through muropeptide analysis cannot be explained in terms of biological function. One example of this is the unknown function of the penta-glycine cross-bridge in *S. aureus*. Another is the unknown reason for some species utilizing lysine and others using diaminopimelic acid to provide the amino group for cross-linking.

We know that some muropeptides are not randomly distributed across the sacculus. D-alanine-D-alanine motifs are associated primarily with new insertion and adopt a range of species specific localizations, as are muropeptides containing artificially introduced D-amino acids after pulse labelling (Kuru *et al*., 2012). It is possible that muropeptide chemistry could mark regions of the sacculus as permissive or restrictive for insertion of new material. For example, a region containing peptides that are either already highly cross-linked or contain only one or two amino acids would not be an efficient acceptor for transpeptidation. In the case of *S. aureus*, the ring/spiral surface architecture is associated with high levels of D-alanine-D-alanine motifs and the knobble architecture with lower levels. Other unknown architectural features are, for example, the orientation of neighbouring glycan chains (parallel, anti-parallel or mixed) and the localization of short/long glycan chains on the sacculus, which is related to the distribution of glycan chain ends.

The peptidoglycan sacculus is an inherited structure, with some regions being several generations old. In *E. coli* and *B. subtilis*, poles are anything between one and in theory a large number of generations old, and in the cylindrical part of the cell old material is dispersed throughout the wall depending on insertion pattern and turnover rate. In *B. subtilis* cables of peptidoglycan are more apparent by AFM imaging on the inside of the sacculus than the outside – attributed to hydrolase activity (Hayhurst *et al*., 2008). If one adopts a planar layered model based on ECT and modelling evidence, again one might expect chemical differences throughout the depth of the wall. Links between chemistry and structure may be particularly important at the poles, which are known to have distinct chemistry (Obermann and Höltje, 1994; de Pedro *et al*., 1997).

In *S. aureus*, orthogonal division leads to ‘sectoring’ of the cell wall with smaller sectors being generally made of older material (Turner *et al*., 2010). It is possible that ageing of peptidoglycan leads to distinct chemistry or conformation as the effects of modifying and hydrolysing enzymes increasingly mask those of synthesis and insertion.

## Peptidoglycan structure – the missing link between chemistry and architecture

Peptidoglycan has been the subject of much controversy over the decades, in some part due to its importance for bacterial life but also because it is the target for some of our most important antibiotics. Advances in understanding have consistently been underpinned by advances in experimentation, such as HPLC and imaging technologies, and further breakthroughs will likely require more technological leaps, most of which will be impossible to foresee!

There are a small but growing palette of fluorescent probes, which enable us to map moieties within the cell wall or isolated sacculus. An expanded repertoire of probes may allow us to address the biological function of aspects of variation in peptidoglycan across species and within the sacculus. Some probes may be combined with super-resolution imaging techniques such as stochastic optical reconstruction microscopy (STORM, Rust *et al*., 2006; Heilemann *et al*., 2008; Turner *et al*., 2013) or structured illumination microscopy (SIM, Gustafsson, 2005; Wheeler *et al*., 2011; Kuru *et al*., 2012) in order to provide functional imaging on length scales similar to those accessible to AFM or ECT. Some of these approaches can be used *in vivo*. AFM and ECT both have demonstrable maximum resolutions far in excess of those which have been achieved in imaging peptidoglycan and further work with these techniques will likely yield more information on the fine structure of peptidoglycan and the way in which strands are arranged to form the larger architectural features, such as ‘piecrusts’ in *S. aureus* and ‘cables’ in *B. subtilis*. Variants of AFM can be used to visualize individual polymer chains in ambient conditions (Mullin and Hobbs, 2011) and even submolecular detail (e.g. the benzene ring structure of pentacene) under high vacuum (Gross *et al*., 2009). These technologies enabling direct imaging of peptidoglycan or imaging of fluorescently labelled constituents at high resolution have huge potential. However, we should not overlook the power of indirect and ensemble measurements, which can be coupled with qualitative and quantitative modelling. Advances in NMR have recently yielded structural details on length scales far smaller than those available to imaging approaches applied to date (Kern *et al*., 2008; 2010,; Sharif *et al*., 2009; Kim *et al*., 2013) and further study with doubtless lead to more insights. Optical microscopy has the distinct advantage of allowing living cells to be studied in reasonably large numbers and this has had an inestimable impact on bacterial cell biology in general. However, optical microscopy can also be used to provide information about cellular mechanical properties; for example by imaging cells growing while embedded in polymers (Tuson *et al*., 2012), in combination with AFM force measurements (Deng *et al*., 2011) or optical trapping (Wang *et al*., 2010). Where the mechanical properties of the peptidoglycan can be successfully isolated from those of other cell components, these techniques afford an opportunity to constrain models of peptidoglycan architectural dynamics *in vivo* that most of the direct imaging techniques cannot.

Peptidoglycan architecture on the scale of tens to hundreds of nm varies between species and within an individual cell. In Gram-negative bacteria AFM and ECT provide some level of access to the underlying structure, and while the agreement between the two techniques is not perfect, there are strong indications that peptidoglycan organization is similar in all Gram-negatives examined so far, albeit with some species specific characteristics. However, in Gram-positives there is at present no way of directly visualizing the interior structure of the sacculus. Here, there is great controversy as authors propose radically different models of internal structure based on the shape or surface features of the sacculus images.

An overarching model of peptidoglycan covering all bacteria is a very appealing prospect, and we have seen that there are statements that can be made about peptidoglycan that apply to all, or to groups of species. However, when one begins to investigate the detail of peptidoglycan chemistry and architecture, diversity is soon revealed and we must be aware of the dangers of over-generalizing. Much of our current data, particularly on the internal structure of Gram-positive peptidoglycan, is indirect, for example in the form of surface images, or population averages. This sort of information puts constraints on the models we can put forward, but sadly does not make it obvious what our models should be. In this imperfect situation, it is critical proposed models are not over-generalized and take into account all available data.

New advances in high resolution imaging technology bring forward the possibility of directly visualizing peptidoglycan chemistry *in situ*. With this type of data we will begin to connect chemistry with architecture and explain the diversity of features that have been observed, yielding further insights into this polymer, which is so important for bacterial life and, through the action of antibiotics, for human health.
